# The Impacts of SARS-CoV-2 Pandemic on Suicide: A Lexical Analysis

**DOI:** 10.3389/fpsyt.2021.593918

**Published:** 2021-02-10

**Authors:** Jucier Gonçalves Júnior, Jair Paulino de Sales, Marcial Moreira Moreno, Modesto Leite Rolim-Neto

**Affiliations:** ^1^Department of Internal Medicine, Santa Casa de Misericórdia de Fortaleza, Fortaleza, Brazil; ^2^Post Graduate Program in Information Sciences (CIn), Federal University of Pernambuco (UFPE), Recife, Brazil; ^3^School of Medicine, Universidade Federal do Cariri (UFCA), Barbalha, Brazil

**Keywords:** coronavirus infections, pandemic, mental health, suicide, qualitative analysis, quantitative analysis

## Abstract

**Background:** Although COVID-19 is a public health emergency, its consequences for the mental health of the population are still scarce. Likewise, its impact on critical situations such as suicide is still poorly explored in the literature. Therefore, this study aimed to analyze in a pioneering way, through lexical and content analysis techniques, the possible impacts of the new COVID-19 pandemic on suicide behavior.

**Methods:** A lexical analysis, whose sample (not probabilistic, i.e., for convenience) was made up of full-length papers (abstracts) and short communications, about suicide behavior in COVID-19 pandemic, in PubMed and Virtual Health Library (VHL) was carried out following a lexical and content analysis using the software IRaMuTeQ, version 0.7 alpha 2.

**Results:** The most frequent active words were suicide behavior (*n* = 649), covid (*n* = 439), health (*n* = 358), mental (*n* = 268), and social (*n* = 220). Four lexical classes were found and organized into two large groups: the first group, formed by the classes 2 (“methods for psychological treatment”) and 3 (“strategies to minimize the COVID-19 impacts”), was the most representative, totaling 50.6% of the text segments and second group formed by classes 1 (“signs of clinical depression”) and 4 (“COVID-19 pandemic as a public health problem”) with 49.4% of the text segments.

**Conclusion:** Facing suicide behavior, the direct effects of the COVID-19 pandemic, and the negative feelings and trigger of previous psychiatric illnesses; the measures to deal with the pandemic such as social isolation, decrease in the number of professionals, the opening hours of health establishments, and decrease in the demand for medications; and competing phenomena such as the spread of fake news and lack of empathy are aggressive and potentiating factors of suicidal ideation.

## Introduction

Around one million deaths by suicide behavior are registered every year (1), which is one person every 40 s and 1.4% of all deaths worldwide, being the 18th leading cause of death in 2016. Thus, suicide behavior is a global phenomenon and occurs throughout the life span (2).

In the face of the new coronavirus (COVID-19), SARS-CoV-2, pandemic that occurred in Wuhan, China, in December of 2019 (3), with more cases and news about people in quarantine (4), the isolation and feeling of loneliness are high. As of December 30, 2020, a total of 80,155,187 cases of COVID-19 have been confirmed in 222 countries with 1,771,128 deaths (5).

During pandemics, the number of people whose mental health is affected tends to be greater than the number of people affected by the infection. Indeed, in a pandemic, fear increases stress, negative symptoms, and anxiety levels in the general population and intensifies the symptoms in individuals with pre-existing psychiatric disorders (6, 7). A Chinese study reported that patients infected with COVID-19 (or suspected of being infected) may experience intense emotional and behavioral reactions, such as fear, boredom, loneliness, anxiety, insomnia, or anger (8) as has been reported about similar situations in the past.

Suicide behavior is likely to become a more pressing concern as the pandemic spreads and has longer-term effects on the general population, as much as on the economy, and on vulnerable groups (9). The increase in suicide behavior rates is being predicted worldwide, from the United States (10) to India (11).

However, as COVID-19 is a new disease, both biological and psychological impacts lack greater theoretical input. And when it comes to suicide behavior in the context of a pandemic, that situation becomes more critical. Therefore, this study aimed to analyze, through lexical and content analysis techniques from recent original publications, the possible impacts of the new COVID-19 pandemic on suicide behavior. Moreover, a lexical analysis could be considered a complementary approach to a thematic analysis once it allows a deeper investigation of textual data (12).

## Methods

A lexical analysis whose sample (not probabilistic, i.e., for convenience) was made up of abstracts in full-length papers and complete texts in short communications in PubMed and Virtual Health Library (VHL), which hosts recognized databases—LILACS (*Literatura Latino-americana e do Caribe em Ciências da Saúde*), MEDLINE, and SciELO (Scientific Electronic Library Online)—was carried out.

### Procedure

Data collection was performed in databases, and the following descriptors were used: #1 “coronavirus infections” (Medical Subject Headings [MeSH term]) AND #2 “suicide” (MeSH), which referred to mental health situations facing suicide or suicidal behavior in the pandemic period of SARS-CoV-2.

### Eligibility Criteria

The period reported in the literature ranged from December 2019 to April 2020 for two phases since the pandemic started in this time span. Compilation of the data was performed in May 2020. The manuscript and abstract selection occurred primarily through the analysis of abstracts (for full-length papers) and short communications. The analysis had the following eligibility criteria: (1) texts written in English, Portuguese, or Spanish; (2) studies addressing the impact of the COVID-19 pandemic on suicide attempt; (3) titles with a combination of search terms; and (4) full text available through the CAPES (Coordination of Personal Improvement of Higher Level) Periodicals Portal, a virtual library created by the Brazilian Ministry of Health where content is restricted to authorized users. Monographs, dissertations, and these were excluded. Manuscripts that were repeated in more than one of the databases were counted only once. Some articles which have already generally approached the suicide behavior in other viruses/pandemics were excluded.

To ensure the trustworthiness of the findings, data collection was performed, individually, by two researchers with divergences being solved by a third senior researcher.

### Data Analysis

After tabulation, the data were processed by using the IRaMuTeQ (*Interface de R pour les Analyses Multidimensionnelles de Textes et de Questionnaires*) software, version 0.7 alpha 2, developed by Pierre Ratinaud, which allows the statistical analysis of textual corpus and individual tables/words (13). IRaMuTeQ provides five types of analysis: classical textual statistics, research regarding group specificities, descending hierarchical rank, similarity analysis, and word cloud (14–16).

The abstracts and short communications were transformed in three corpus named by the letter “S” and analyzed through similarity analysis, word cloud, confirmatory factorial analysis (CFA), and descendent hierarchical classification (DHC) (14, 15).

Before the analysis was started, in the text segment parameters, only “full” language elements were selected as assets, adjectives, unrecognized forms, nouns, verbs, and auxiliary nouns and verbs such as complementary (supplementary), aiming to enrich the text content.

### Ethical Issues

Since this is a qualitative study with public domain information, Resolution 510/16 of the Brazilian National Health Council (CNS) ensures its exemption from submission to a Human Beings Research Ethics Committee. The ethical criteria of the Declaration of Helsinki and international standards were observed.

## Results

According to the research strategy in PubMed, 54 papers were found. Thirty-nine articles were selected after applying the filters. Fifteen were out of context and/or beyond the search period. In VHL, 29 articles were found, but none was selected since 12 were repeated and the 17 others were out of context.

Initially, the corpus was built with 39 texts separated by 1,184 text segments, in which only 885 were used (74.75%). The lexicographic analysis of the textual corpus produced 42,562 occurrences (words and forms), with 3,195 being active forms. The most frequent active words were suicide (*n* = 649), covid (*n* = 439), health (*n* = 358), mental (*n* = 268), and social (*n* = 220).

**Figure 1:**
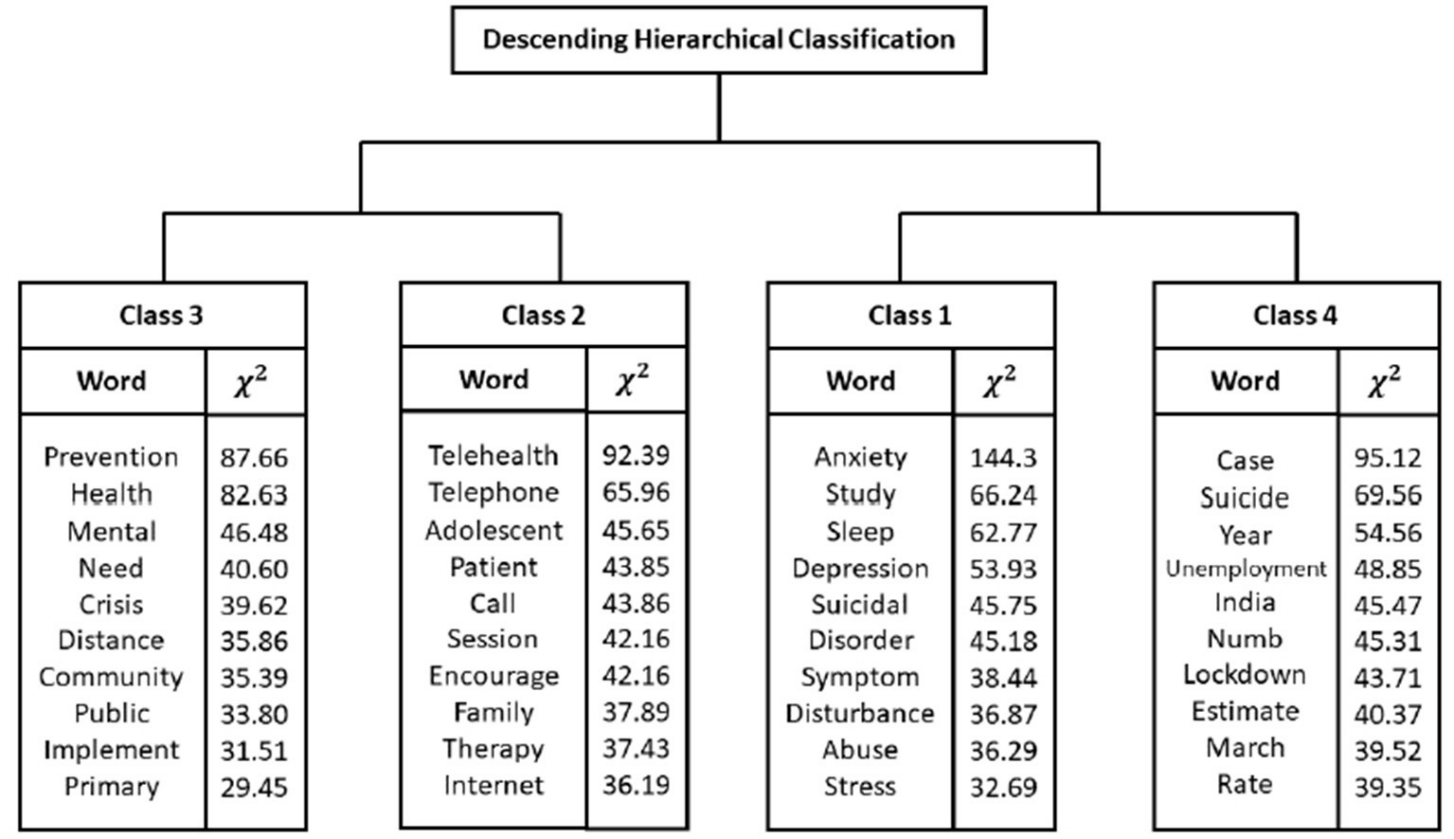
Descending hierarchical classification (dendrogram) with most frequently words and chi-square values.

Moreover, the DHC of active words produced five lexical classes divided into two major groups, shown in the dendrogram (Figure 1). The first group, formed by classes 2 and 3, was the most representative, totaling 50.6% of the text segments. Complementarily, the second group was formed by classes 1 and 4 (49.4% of the text segments). The four lexical classes received the following names: “signs of clinical depression” (class 1); “methods for psychological treatment” (class 2); “strategies to minimize the COVID-19 impacts” (class 3); and “COVID-19 pandemic as a public health problem” (class 4).

**Figure 2:**
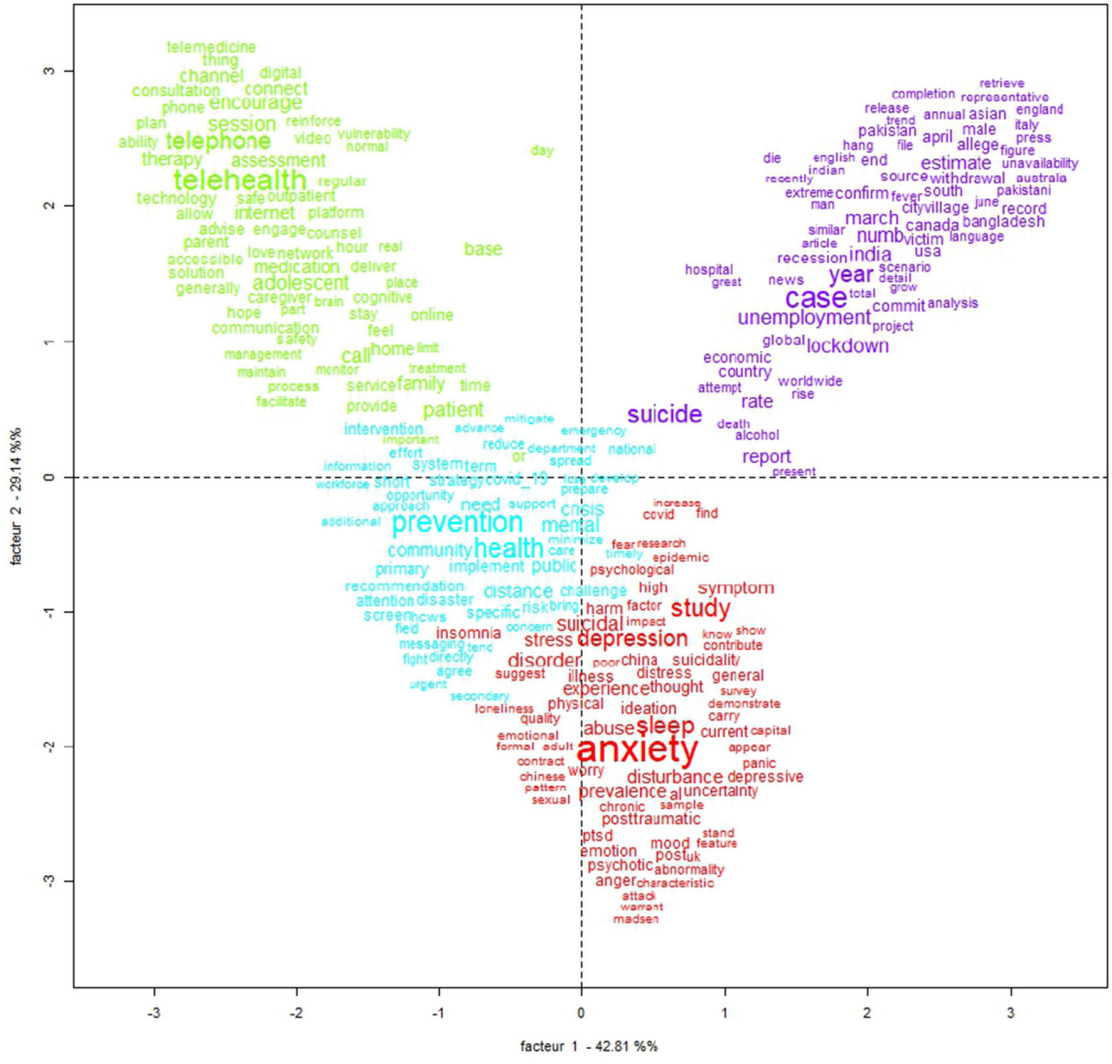
Correspondence factorial analysis (CFA) (IRaMuTeQ).

Regarding the CFA, two factors explain 42.81% and 29.14% of the model, which are represented on the X and Y axes in Figure 2. The two-dimensional view clearly presents four distinct areas, which are directly associated with the four classes previously presented.

**Figure 3:**
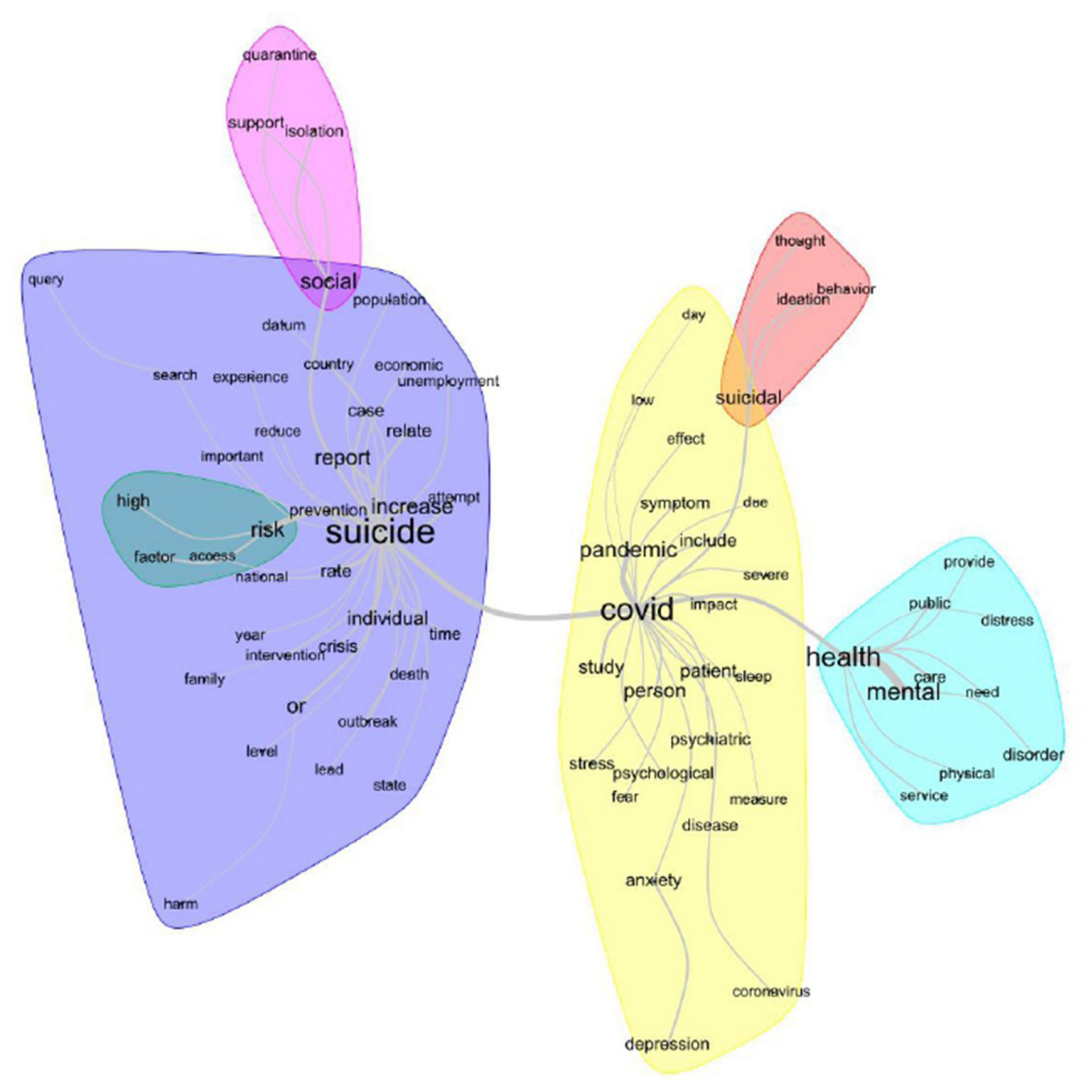
Lexical similarity analysis (IRaMuTeQ).

Figure 3 shows the similitude analysis, using a graph that represents the connection between words in the analyzed textual corpus (17). In this case, six groups of words were constructed. The first DHC class, “signs of clinical depression,” seems to be related to the yellow and red groups of words. Further, “methods for psychological treatment” (class 2) and “strategies to minimize the COVID-19 impacts” (class 3) seem to be related to the purple group. Finally, the fourth class (“COVID-19 pandemic as a public health problem”) and the blue group have words with close meanings.

## Discussion

The most frequent active words were suicide (*n* = 649), covid (*n* = 439), health (*n* = 358), mental (*n* = 268), and social (*n* = 220). In DHC, four lexical classes emerged (Figure 1). The second (“methods for psychological treatment”) and third (“strategies to minimize the COVID-19 impacts”) classes were the most representative, totaling 50.6% of the text segments, and are correlated to each other (Figures 1, 2). The most evoked words in the second class (“strategies to minimize the COVID-19 impacts”) are prevention, health, mental, need, crisis, and distance (Figures 1, 3).

Indeed, with the COVID-19 pandemic, the World Health Organization (WHO) affirms that introducing quarantine measures early may delay the introduction of the disease to a country or area or may delay the peak of an epidemic in an area where local transmission is ongoing or both (7, 18). Therefore, the quarantine has an impact on worsening mental and biologic health and causes strong emotions in adults and children (7, 19). It enhances insomnia, denial, stress, anxiety, fear, depressive symptoms, and anger (20) (words evoked in the first class) and increases use of tobacco, alcohol, or other drugs (19).

In the second great group, with 49.6% of the text segments each, are the first (“signs of clinical depression”) and fourth (“COVID-19 pandemic as a public health problem”) classes. The first class evokes the words anxiety, study, sleep, depression, and suicidal (Figures 1, 3). In this context, it is understandable that the correlations established in the first class are evident, since it correlates anxiety and mood disorders (such as depression) and their consequences, such as insomnia, with the increase of suicide risk. In this case, the literature affirms that during and following the COVID-19 outbreak and the outcomes of isolation and quarantine, we might see an increase in suicidal ideation and behavior among at-risk populations (21). This could happen due to the factors mentioned above, associated with the financial crisis (22), decreased support from health services, limited access to medications (3, 4), and bullying in those who are infected and who have previous psychiatric comorbidities.

The correlation in the first and third classes reminds us that suicide behavior is a public health problem and demands attention from governments, public agencies, and health professionals. Estimates suggest that fatalities could rise to 1.5 million by 2020 (23). In the United States, suicide is one of the leading causes of death among young people. It is the third leading cause of death among 15–24-year-olds and the second leading cause of death among 25–34-year-olds (24). In the Western Pacific Region, it accounts for 2.5% of all economic losses due to diseases. In most European countries, the number of suicides is larger than annual traffic fatalities (18).

The second class (“methods for psychological treatment”), when the evoked words are analyzed, calls us to action. It is mandatory to emphasize social support for populations and patients in the COVID-19 pandemic, reinforcing that there is always hope and that there are several solutions to any problem (21); to improve self-esteem and social bonds, especially with family and friends, by having social support, being in a stable relationship, and having religious or spiritual commitment (18); to monitor dysphoric mental states such as irritability and aggression and to not disseminate information from unofficial sources (6). For governments, it is important to standardize psychotropic medications and make them available; to conduct training in stress management, trauma, depression, and risk behavior protocols; to provide alternative service channels (apps, websites, and telephones) (25, 26); and to encourage the participation of multidisciplinary mental health teams at national, state, and municipal levels (27, 28). Finally, the words highlight the fundamental role of social networks and telemedicine as a tool for health care, monitoring, and health promotion during the COVID-19 pandemic against suicide behavior and psychiatry illness. As shown in Figure 2, the most prominent words in the green group are encourage, telehealth, and telephone.

The fourth class (“COVID-19 pandemic as a public health problem”) evokes the word unemployment (fourth most common word in this class). Truly, suicide behavior has many complex underlying causes, including poverty and unemployment (23), especially harming the extremes of age like the elderly and children in a pandemic such as COVID-19. A study with forecasting in 63 countries observed that suicide risk was elevated by 20–30% when associated with unemployment during 2000–2011 (including the 2008 economic crisis) (29), and in New Zealand, 2.04 million cases are included to investigate the relation between suicide and unemployment. The authors concluded that unemployment was associated with a 2 to 3 fold increased relative risk of death by suicide associated to 18–24-year-old men, compared with being employed (30). It is important to consider that in situations of humanitarian crisis such as a pandemic, the economic impact of readjustments in public accounts, the collapse of industry/commerce, and the increase in the prices of basic inputs worsen the scenario (3).

## Final Considerations

This study aimed to carry out in a pioneering way a lexical analysis of recent publications on the relationship between the COVID-19 pandemic and suicide behavior. Therefore, a search for original papers was conducted. Then, based on the findings, a lexical analysis was performed using the IRaMuTeQ software.

During a humanitarian crisis, it is not uncommon for the mental health of the general population and, above all, for psychiatric patients to be neglected. From this discursive textual analysis, this reality seems apparent. Thus, a collective effort involving civil society, governments, and medical entities to develop urgent damage control/health promotion policies is of utmost importance.

The mapping of risk factors for suicidal behavior, such as patients with previous psychiatric illnesses and/or negative feelings; social isolation and competing phenomena such as the spread of fake news and lack of empathy; poverty; precarious health care conditions or their absence (decrease in the number of professionals, the insufficient opening hours of health establishments, and decreased availability of medications for continuous use); and structured actions to reduce these situations, is essential so that there is no new pandemic within COVID-19.

## Data Availability Statement

The raw data supporting the conclusions of this article will be made available by the authors, without undue reservation.

## Author Contributions

All the authors designed the review, developed the inclusion criteria, screened titles and abstracts, appraised the quality of included papers, and drafted the manuscript.

## Conflict of Interest

The authors declare that the research was conducted in the absence of any commercial or financial relationships that could be construed as a potential conflict of interest.
